# State dependent effects on the frequency response of prestin’s real and imaginary components of nonlinear capacitance

**DOI:** 10.1038/s41598-021-95121-4

**Published:** 2021-08-09

**Authors:** Joseph Santos-Sacchi, Dhasakumar Navaratnam, Winston J. T. Tan

**Affiliations:** 1 Surgery (Otolaryngology), Yale University School of Medicine, BML 224, 333 Cedar Street, New Haven, CT 06510 USA; 2 Neuroscience, Yale University School of Medicine, 333 Cedar Street, New Haven, CT 06510 USA; 3 Neurology, Yale University School of Medicine, 333 Cedar Street, New Haven, CT 06510 USA; 4 Cellular and Molecular Physiology, Yale University School of Medicine, 333 Cedar Street, New Haven, CT 06510 USA

**Keywords:** Biophysics, Neuroscience, Physiology

## Abstract

The outer hair cell (OHC) membrane harbors a voltage-dependent protein, prestin (SLC26a5), in high density, whose charge movement is evidenced as a nonlinear capacitance (NLC). NLC is bell-shaped, with its peak occurring at a voltage, V_h_, where sensor charge is equally distributed across the plasma membrane. Thus, V_h_ provides information on the conformational state of prestin. V_h_ is sensitive to membrane tension, shifting to positive voltage as tension increases and is the basis for considering prestin piezoelectric (PZE). NLC can be deconstructed into real and imaginary components that report on charge movements in phase or 90 degrees out of phase with AC voltage. Here we show in membrane macro-patches of the OHC that there is a partial trade-off in the magnitude of real and imaginary components as interrogation frequency increases, as predicted by a recent PZE model (Rabbitt in Proc Natl Acad Sci USA 17:21880–21888, 2020). However, we find similar behavior in a simple 2-state voltage-dependent kinetic model of prestin that lacks piezoelectric coupling. At a particular frequency, F_is_, the complex component magnitudes intersect. Using this metric, F_is_, which depends on the frequency response of each complex component, we find that initial V_h_ influences F_is_; thus, by categorizing patches into groups of different V_h_, (above and below − 30 mV) we find that F_is_ is lower for the negative V_h_ group. We also find that the effect of membrane tension on complex NLC is dependent, but differentially so, on initial V_h_. Whereas the negative group exhibits shifts to higher frequencies for increasing tension, the opposite occurs for the positive group. Despite complex component trade-offs, the low-pass roll-off in absolute magnitude of NLC, which varies little with our perturbations and is indicative of diminishing total charge movement, poses a challenge for a role of voltage-driven prestin in cochlear amplification at very high frequencies.

## Introduction

Prestin (SLC26a5) is a membrane-bound voltage-dependent protein^1,2^, whose activity drives outer hair cell (OHC) electromotility (eM)^3^, the latter yet considered to provide for cochlear amplification at very high acoustic frequencies (50–100 kHz). Evidence supporting high frequency electromotility stems from whole-cell microchamber experiments and mechanical measures in vivo during electrical stimulation within the organ of Corti^4–6^. Nevertheless, recent observations against this concept have been made, indicating lower pass behavior^7–11^. Regardless, the voltage dependent activity of prestin itself has been well characterized over the years^12,13^, typically analyzed by evaluating membrane capacitance (δQ/δV) (i.e., NonLinearCapacitance, NLC). The proteins’ aggregate sensor charge displays a sigmoidal Boltzmann (Q/V) relationship, being quantitatively characterized by V_h_, the voltage at half maximal charge redistribution across the membrane (near − 40 mV); *z*, the apparent unitary charge (near 0.75); and Q_max_ the maximum charge of a given protein population within the membrane. The parameter *z* is alternatively viewed as a voltage sensitivity, providing a slope factor of the Q/V function (near 35 mV), which indicates a very shallow voltage dependence. Thus, the full range of prestin’s voltage responsiveness spans over 300 mV. Of course, prestin is not unique in generating NLC, as channels whose voltage sensors produce displacement currents show similar behavior^14^.

Prestin has been deemed a piezoelectric (PZE) protein since Iwasa discovered tension sensitivity of NLC under whole cell (WC) voltage clamp^15^. Indeed, prestin’s piezoelectric coefficient is extraordinary, estimated to be four orders of magnitude greater than man made elements^16^. Under WC voltage clamp, V_h_ shifts rightward and peak NLC decreases as membrane tension increases^15,17,18^. In membrane patches, however, peak NLC (or Q_max_) does not alter despite substantial shifts in V_h_^7,19^. The likely reason why apparent Q_max_ is decreased in WC mode is because tension delivery is inhomogeneous^19^, and differentially impinges on multiple independent micro domains of prestin^20^. The disparate summation in WC measures of NLC therefore shows changes in apparent *z* resulting in a reduced peak NLC. Again, the reduction in total charge with tension is not a characteristic of prestin itself, as membrane patch studies clearly show.

Recently, Rabbitt^21^ suggested that the PZE nature of prestin provides power to the cochlear amplifier that correlates with the imaginary component of NLC, that component of charge movement that alters phase in response to viscous load. Such modelled behavior of prestin sensor charge movement, which for a piezo component is coupled to imposed stress, was predicted to account for the apparent incongruity of prestin’s low-pass roll-off in the absolute magnitude of complex NLC^7^ and the expected high frequency behavior of prestin required by cochlear modelers to account for high frequency cochlear amplification. Here we thoroughly evaluate the real and imaginary components of NLC in OHC macro-patches and find partially reciprocal (not of equal magnitude) trade-offs in those components across frequency that coincide with Rabbitt’s model results. We do not use the word reciprocal to intimate any reciprocity between electrical and mechanical interactions, since we made no simultaneous mechanical measures during our patch recordings, notwithstanding the general consensus that displacement currents, hence NLC, signify movements of charged residues in voltage dependent proteins^22^. However, we did previously explore interactions between electrical and mechanical behavior in whole OHCs^8^. Despite the similarity between the PZE model’s complex NLC behavior and our data, we also find that a simple 2-state kinetic model lacking piezoelectric coupling provides similar trade-offs in complex components across frequency. In our biophysical data, we also observed significant effects of initial state, characterized by initial V_h_, on the frequency response of the reciprocal trade-offs. Furthermore, membrane tension differentially alters that frequency response depending on initial state. Whether such observations are in line with PZE theory remain to be determined.

## Results

Rabbitt^21^ modeled prestin and its NLC as a piezoelectric process based on first principles. NLC can be deconstructed into real and imaginary components^23^, since it is defined as the frequency-dependent membrane admittance (Y*_m_(ω)) divided by *iω*, where $\omega =2\pi f \,\text{and}\, i=\sqrt{-1}$,

$${C}_{m}^{*}\left(\omega \right)= \frac{{Y}_{m}^{*}\left(\omega \right)}{i\omega }= \frac{{B}_{m}^{*}\left(\omega \right)}{\omega }-i \frac{{G}_{m}^{*}\left(\omega \right)}{\omega },$$

providing a real capacitive component and an imaginary conductive component. On removing stray capacitance (by subtraction, see “Methods” section) and linear capacitance (by Boltzmann fitting, see “Methods” section) from ${C}_{m}^{*}\left(\omega \right),$ prestin-associated complex NLC(ω) is obtained.

With his PZE model, Rabbitt predicted that the imaginary component of complex NLC should increase over frequency, as the real component decreases. We previously reported on the real and imaginary components of complex NLC^7^ and concluded that the imaginary component was small and essentially frequency independent. However, we did not fit both complex components to Boltzmann functions to unambiguously extract details of those components. In that paper, we focused our attention on the absolute magnitude of complex NLC, i.e., $\sqrt{Re{\left(NLC\right)}^{2}+Im{\left(NLC\right)}^{2}}$, which is comparable to all previous measures of OHC/prestin NLC.

Here we explore in detail both components in 25 macro-patches of the OHC lateral membrane, first under zero pipette pressure, namely in the absence of externally applied membrane tension. For the 3D plots, black dots depict average data, and magenta dots depict standard error (+ SE). The multicolored shading is provided by the *interp* surface plot function in Matlab, and the red and blue solid lines depict Boltzmann fits (see “Methods” section) at selected frequencies.

**Figure 1:**
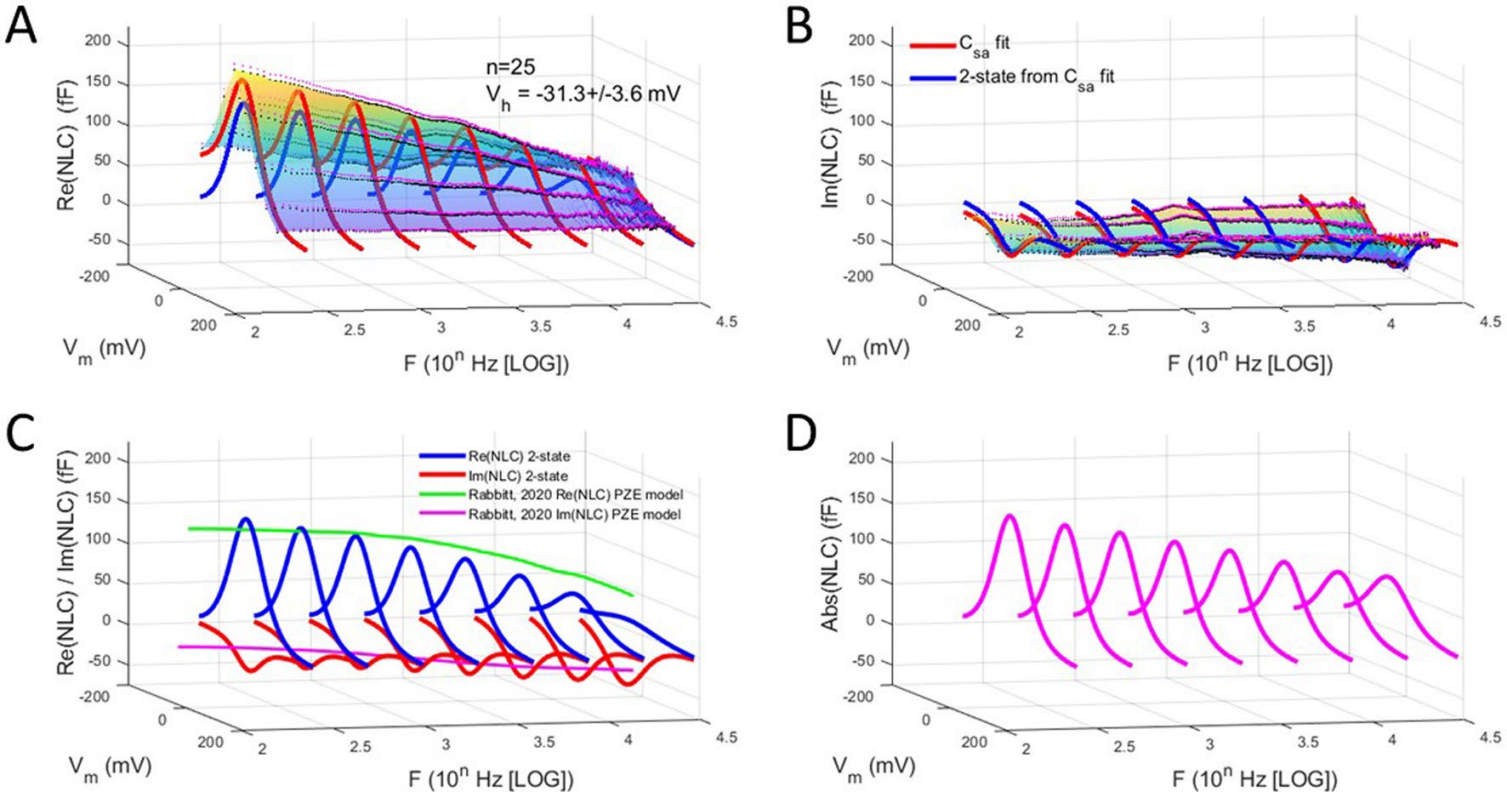
Real and imaginary components of patch NLC. **(A)** Real component of complex NLC across interrogating frequency. Note continuous decrease as frequency increases. Fits are made to Eq. (1) to extract the 2-state (blue line) response at selected frequencies. V_h_ is average between 0.244 and 2.44 kHz. **(B)** Imaginary component of complex NLC. In this case, magnitude increases with frequency, but there is not a full reciprocal trade-off with the real component. **(C)** Plot of the 2-state fits across frequency. Superimposed is the PZE model response at V_h_ of Rabbitt (2020), which shows general agreement within this bandwidth. **(D)** Plot of the absolute magnitude of NLC, namely, $\sqrt{Re{\left(NLC\right)}^{2}+Im{\left(NLC\right)}^{2}}$, indicating a continuous reduction of prestin activity across frequency.

Figure 1 shows that indeed, Rabbitt’s modelling prediction concerning our data that he references is borne out. There appears to be a trade-off between real (Fig. 1A) and imaginary (Fig. 1B) component peak magnitude across frequency, especially revealed in the extracted 2-state fits of NLC (Fig. 1C). Superimposed lines in green and magenta are the PZE model responses at V_h_ from Rabbitt^21^, which shows general agreement within this bandwidth. Figure 1D shows the absolute magnitude of NLC that continuously decreases across frequency. Thus, while there are reciprocal trade-offs between the real and imaginary NLC components, the trade-off is not fully reciprocal; that is, the overall kinetics of prestin (governed by a host of molecular loads) slows.

**Figure 2:**
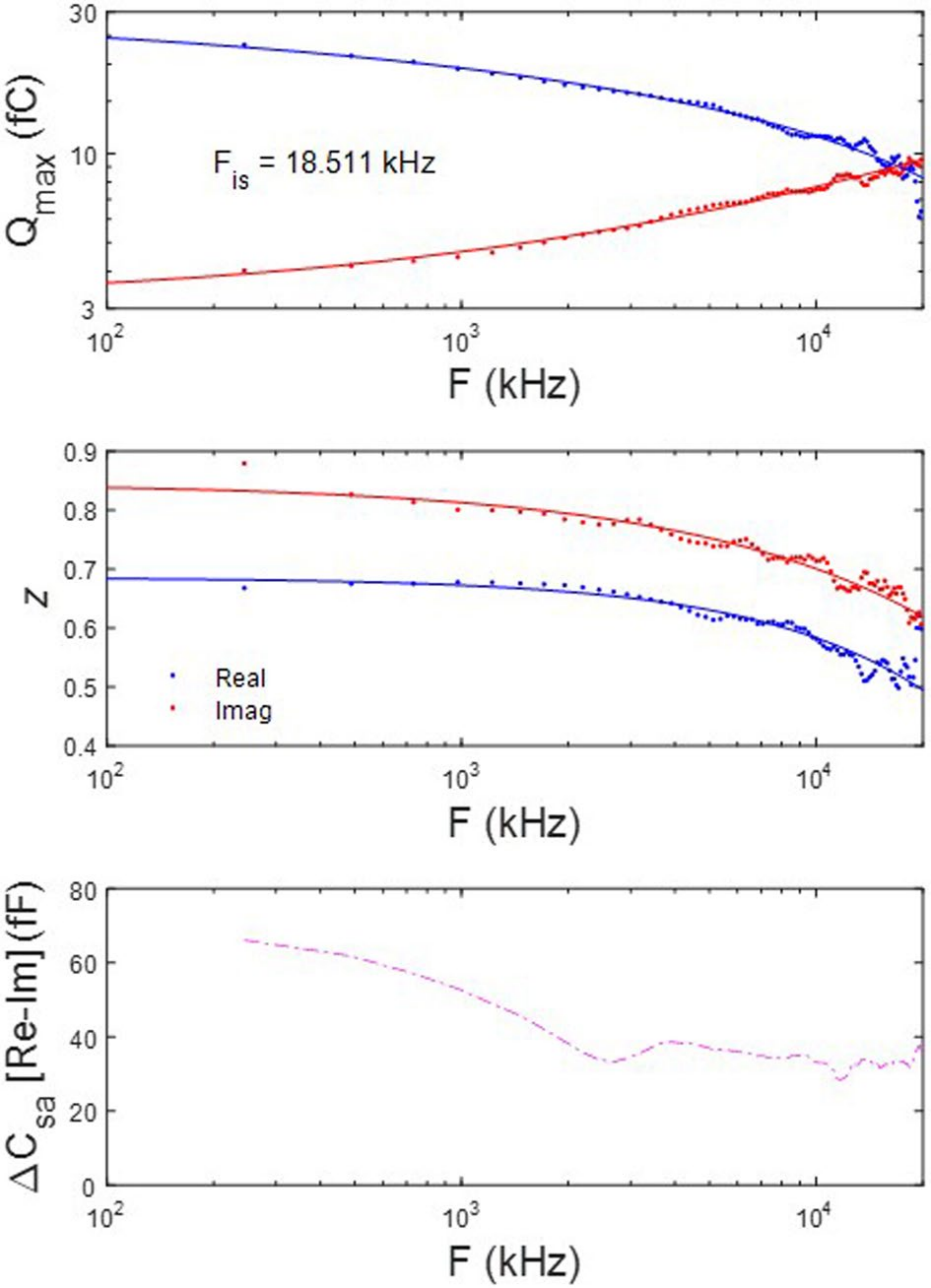
Frequency dependence of complex NLC components. *Top panel* Plot of fitted Q_max_ of 2-state components of complex NLC. The real and imaginary component magnitudes intersect at a particular frequency, F_is_. Here the imaginary component absolute magnitude is plotted. Fits (solid lines) are with Eq. (2). Red is real component; blue is imaginary component. F_is_ is 18.5 kHz. *Middle panel* Plot of the Boltzmann parameter, *z*. Both decrease in parallel with frequency, with the imaginary component being smaller, indicating a shallower voltage sensitivity. *Bottom panel* Plot of difference between real and imaginary ΔC_sa_, showing some low frequency dependence. Figure 1A,B indicate that ΔC_sa_ is largely absent in the imaginary component.

Figure 2 (*top panel*) highlights the relationship between real and imaginary components of NLC. Here we plot extracted Q_max_ from fitted NLC components across frequency. The trade-off in component magnitude shows that at a particular frequency (F_is_), the two intersect, and above that frequency the imaginary component will dominate. Rabbit^21^ modeled that the imaginary component will eventually reduce to zero after peaking, but here (because of our limited frequency interrogation range) we use F_is_ to explore influences on NLC frequency response (see below). Figure 2 (*middle panel*) also shows that the voltage sensitivity (*z*) of the two components decreases in parallel across frequency, possibly indicating an increasingly impeded sensor charge movement within the membrane plane. In other words, voltage-induced conformational switching of prestin is depressed as frequency increases.

Another interesting observation that this fitting exercise exposes relates to the offset (ΔC_sa_) in capacitance at negative voltages, which is uniformly observed in all previous studies reporting on OHC NLC. Indeed, when we first observed this offset and developed the method used to fit the response^24^, we thought it was solely due to surface area alterations as prestin changed states from expanded to contracted. Surface area change in prestin embedded membrane was first reported by Kalinec et al.^25^. However, the magnitude of ΔC_sa_ is greater than that expected for surface area changes alone in prestin. Thus, it likely includes changes to overall dielectric properties of the membrane, as well. Figure 2 (*bottom panel*) compares the offsets in real and imaginary component fits by plotting the difference between the two. The magnitude of ΔC_sa_ is little affected across frequency, and, in fact, it can be seen in Fig. 1A,B that the offset is virtually absent in the imaginary component of NLC. Thus, the charge movement associated with the offset is solely capacitive in nature.

**Figure 3:**
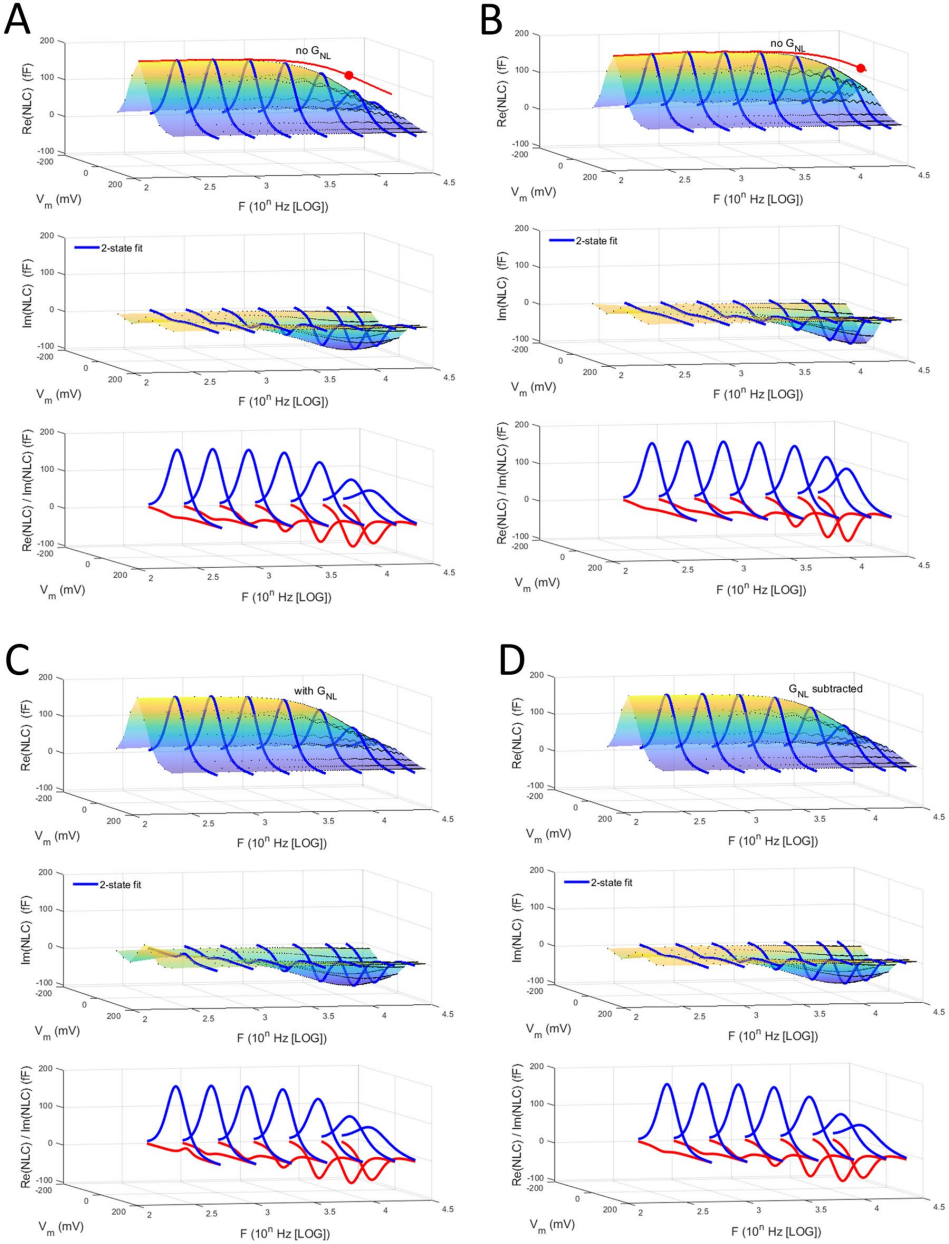
Real and imaginary components of NLC generated by a solely voltage-dependent 2-state kinetic model (see “Results” section for details), with characteristic cut-offs at V_h_ dependent on transition rates, α and β. **(A)** simulations with both rate constants (α_0_ = β_0_) equal to 45,000/s, and **(B)** with both rate constants equal to 90,000/s. In addition to the complex component plots, the absolute magnitude of NLC at V_h_ is shown with red lines. The rates provide characteristic time constants (tau = 1/(α_0_ + β_0_); 11.1 μs and 5.6 μs, respectively) for charge movement in the time domain at the potential V_h_. In the frequency domain, the cut-off frequency at V_h_ is defined as F_c_ = 1/(tau × 2π), 14.3 and 28.6 kHz, respectively. The filled red circles mark the cut-off frequencies. **(C)** Same model as in **(A)**, but with an additional nonlinear DC conductance (designed to be like that in our patch data) which distorts the low frequency response of the imaginary component. **(D)** After removing the DC conductance with the approach detailed in the “Methods” section, the response is essentially the same as in **(A)**, where no DC conductance is included, confirming the validity of our approach to remove any residual DC conductance in our patch data.

Do these real and imaginary component trade-off observations necessarily indicate that prestin is piezoelectric? Figure 3 shows that the same basic behavior observed in our data is recapitulated by a simple 2-state kinetic model with voltage-dependence only, i.e., both real and imaginary NLC components are observed, with trade-offs in magnitude across frequency.

This simple 2-state model for charged prestin particles possesses an expanded (X) and compact (C) state, with the population of prestin molecules redistributing during changes in membrane voltage.

$${\text{X}}\underset{\upbeta }{\overset{\upalpha }{\rightleftharpoons}}{\text{C}}.$$

The forward and backward rates (α, β, respectively) are governed solely by changes in membrane voltage (V_m_) about a characteristic potential, V_h_, where particles (charges) are distributed equally on either side of the membrane field. *z* denotes the voltage sensitivity or unitary particle charge (e^-^ X distance travelled perpendicular to the membrane field). F, R and T have their usual meanings.

$$\alpha = {\alpha }_{0} \text{exp}\left(\frac{zF\left({V}_{m}-{V}_{h}\right)}{2RT}\right),$$

$$\beta = {\beta }_{0}\text{exp}\left(\frac{zF\left({V}_{m}-{V}_{h}\right)}{2RT}\right).$$

In Fig. 3A we show results from simulations with both rate constants (α_0_ = β_0_) equal to 45,000/s, and in Fig. 3B equal to 90,000/s. V_h_ was set to − 40 mV. These rate constants provide characteristic time constants (tau = 1/(α_0_ + β_0_)) for charge movement in the time domain at the potential V_h_. In the frequency domain, the magnitude response is a single Lorentzian (equivalent to a first-order RC response, whose transfer function displays the same complex behavior); the cut-off frequency at V_h_ is defined as F_c_ = 1/(tau × 2π), where the response falls to 0.707 of the initial low frequency magnitude (or correspondingly where a phase lag of 45 degrees develops). In Fig. 3A,B, in addition to the complex component plots, the absolute magnitude of NLC at V_h_ is shown with red lines, and the red circles mark the measured cut-off frequency, which agrees with that calculated from the utilized rate constants. The real component of complex NLC is lower pass than the absolute value. Thus, it is the absolute value of NLC that appropriately characterizes the model’s frequency response, and also that of the biophysical data. We have previously used the absolute magnitude of complex NLC to characterize prestin frequency response in macro-patches^7^.

Additionally, in Fig. 3 we illustrate the validity of our method of subtraction of a nonlinear DC conductance from our patch admittance data prior to subsequent analysis. With a nonlinear DC conductance included in the model (Fig. 3C), like that we find in membrane patches, the imaginary component at low frequencies is distorted. Following removal (Fig. 3D), complex NLC is equivalent to the model without such conductance (Fig. 3A). In the 2-state model, overall NLC behavior arises due to the exponential voltage-dependent transition rates between prestin’s expanded and contracted states. Altering the rate constants will shift the cut-off and the component magnitudes (compare Fig. 3A,B), like alterations of the time constant for an RC response. There is no intrinsic piezoelectric coupling between voltage and tension embodied in this simplest 2-state model, nor in a first order RC circuit. But, without the delays introduced in those conformational transitions (i.e., in a simple ultra-fast 2-state model), no imaginary component exists within our interrogation bandwidth. Thus, the requirement of piezoelectricity in prestin is not firmly established by the trade-off in real and imaginary components of NLC that we find across stimulus frequency.

The data we have presented thus far are from averaged NLC, but we know that NLC V_h_ can vary among cells (and patches) due to a variety of reasons, e.g., membrane tension^15,17,18^, anions^26–28^, temperature^29–31^, initial voltage conditions^32,33^, and membrane cholesterol content^34,35^. Indeed, prestin likely presents characteristics that vary along the length of the cell^20^, where functional expression (e.g., V_h_) can be non-homogenous^36,37^. Consequently, we sought to characterize the dependence of NLC real and imaginary component behavior on prestin’s initial state; namely, as typified by initial V_h_, which reports on the distribution of proteins in either the expanded or contracted states. First, we categorized NLC data into groups possessing V_h_ values above and below − 30 mV.

**Figure 4:**
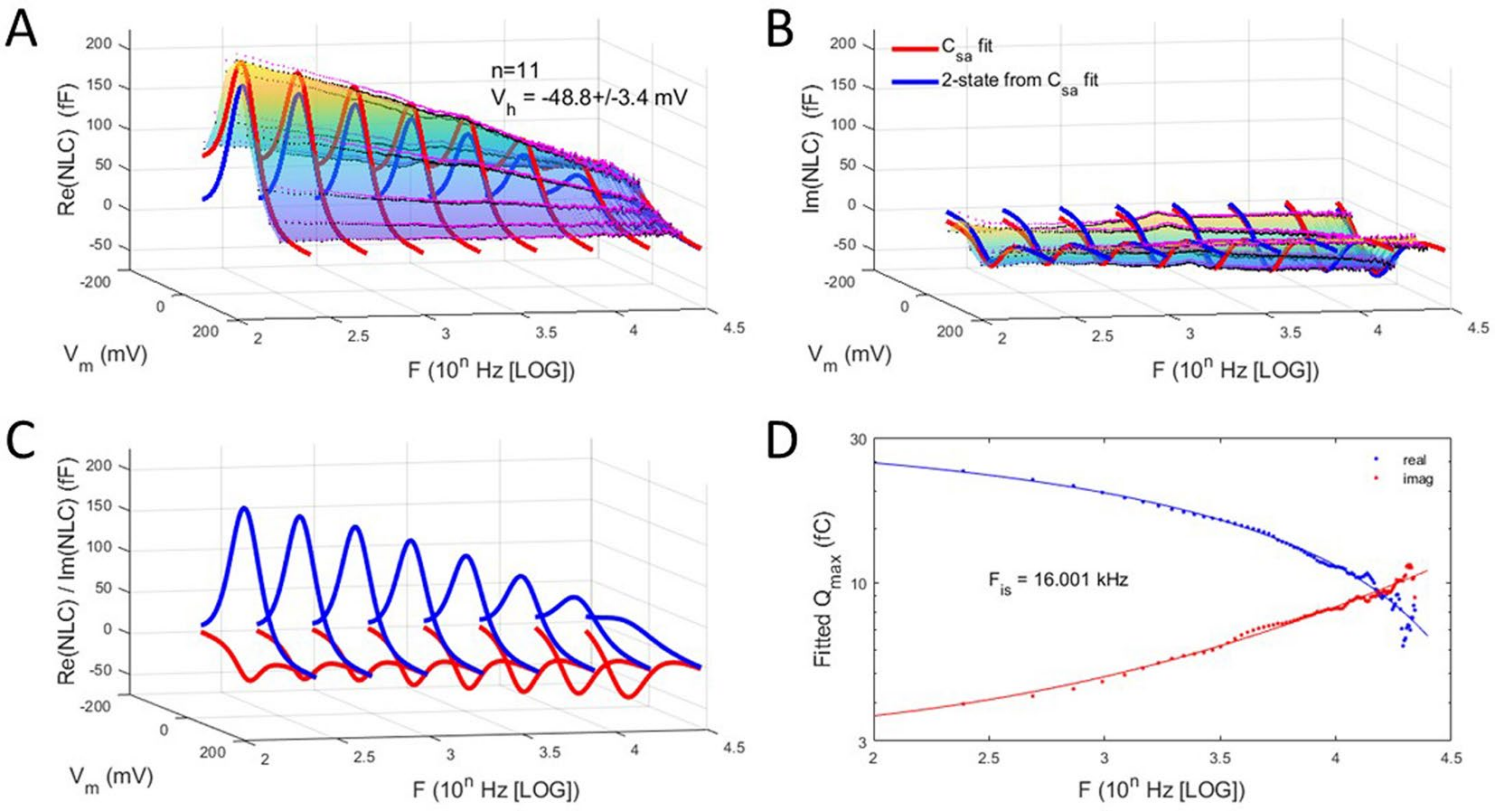
Complex NLC patch data for patches categorized with V_h_ more negative than − 30 mV. **(A–C)** Legends to Fig. 1A–C analogously apply here. **(D)** Plot of fitted Q_max_ of 2-state components of complex NLC. The real and imaginary component magnitudes intersect at a particular frequency, F_is_. Here the imaginary component absolute magnitude is plotted. Fits (solid lines) are with Eq. (2). Red is real component; blue is imaginary component. F_is_ is 16 kHz.

**Figure 5:**
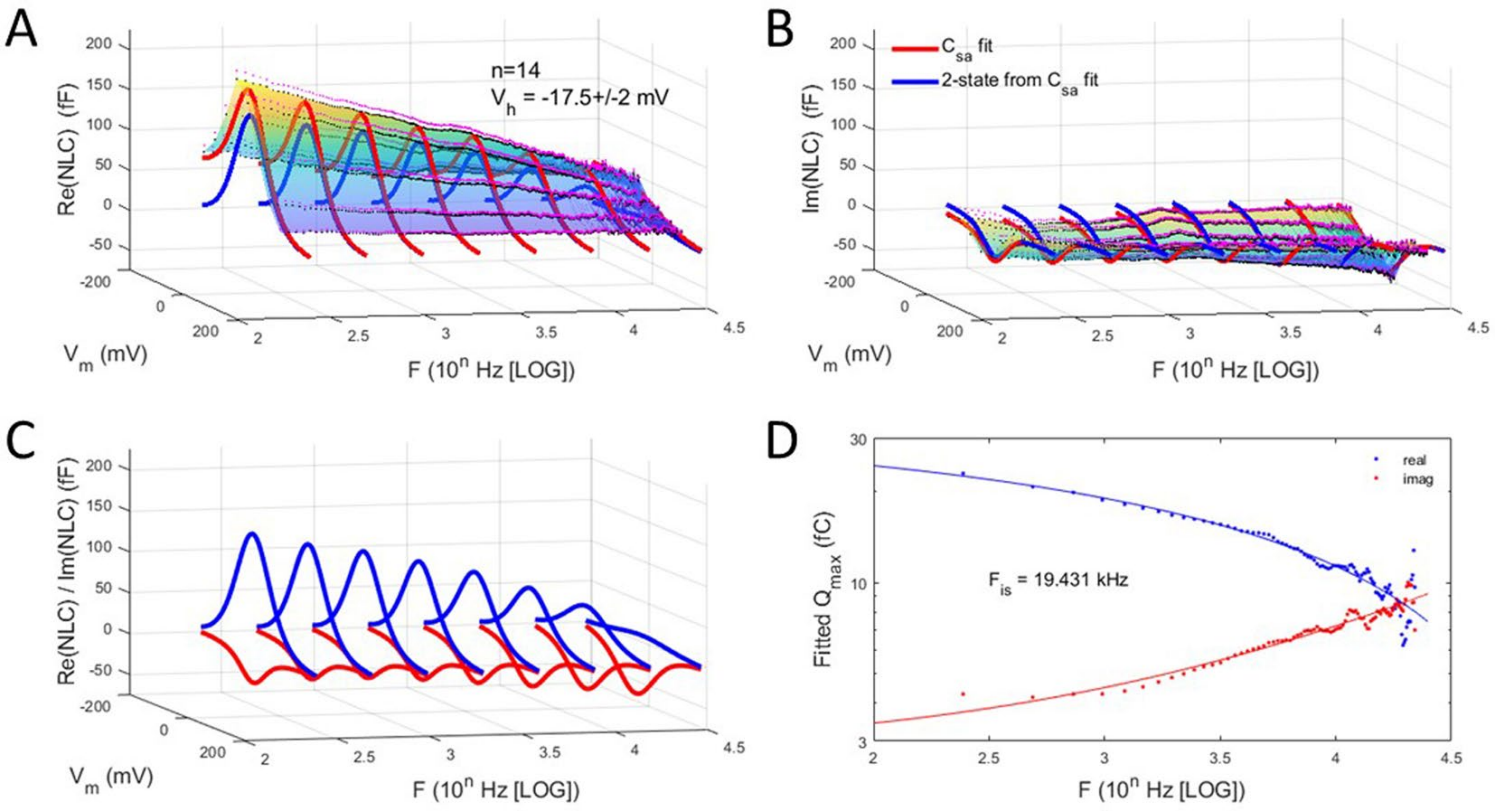
Complex NLC patch data for patches categorized with V_h_ more positive than − 30 mV. **(A–C)** Again, legends to Fig. 1A–C analogously apply here. **(D)** Plot of fitted Q_max_ of 2-state components of complex NLC. The real and imaginary component magnitudes intersect at a particular frequency, F_is_. Here the imaginary component absolute magnitude is plotted. Fits (solid lines) are with Eq. (2). Red is real component; blue is imaginary component. F_is_ is 19.4 kHz.

**Figure 6:**
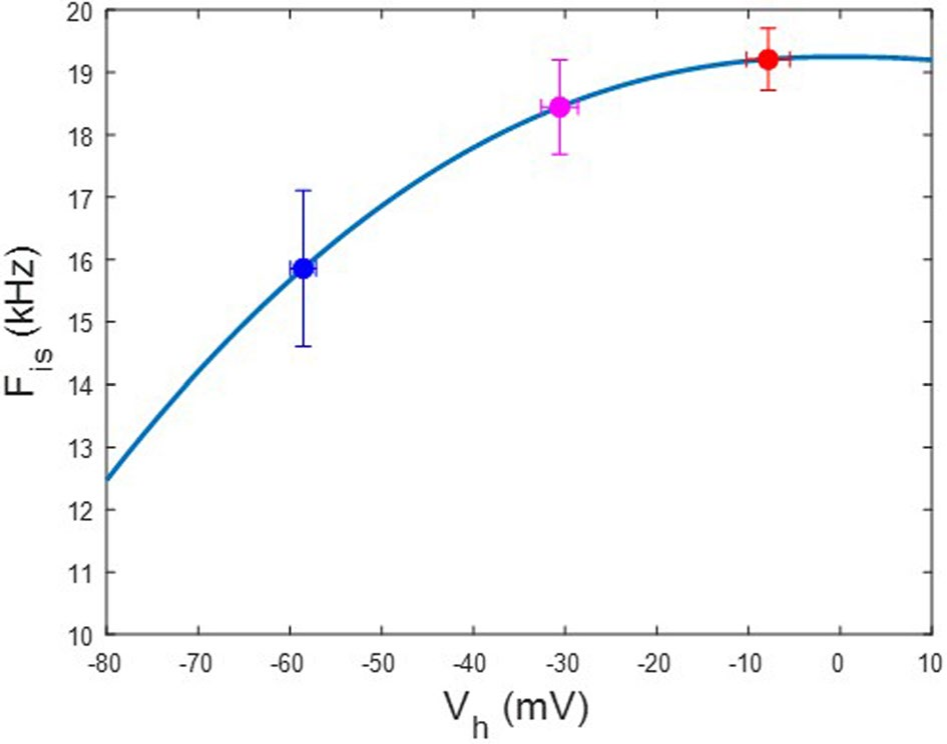
F_is_ for complex NLC patch data for patches recategorized into 3 V_h_ groups. Error bars are SE. Note that as V_h_ is more negative, F_is_ increases nonlinearly. This shows that V_h_ influences the frequency responses of real and imaginary components of complex NLC.

Figures 4 and 5 show these categorized averaged responses. Means of V_h_ for the two groups are − 48.8 ± 3.4 mV (n = 11) for the negative range group, and − 17.5 ± 2 mV (n = 14) for the positive range group. Differences between the frequency response of the two groups are apparent, especially in imaginary components (Figs. 4A–C, 5A–C). These differences are highlighted by plotting the intersection of the fitted Q_max_ magnitudes of each NLC component (Figs. 4C, 5C), where the intersection frequency (F_is_) is at a lower frequency for the negative range group (16 kHz) than the positive range group (19.4 kHz). Figure 6 recategorizes V_h_ into 3 groups, and clearly shows a nonlinear increase of the intersection frequency with more depolarized initial V_h_ conditions.

**Figure 7:**
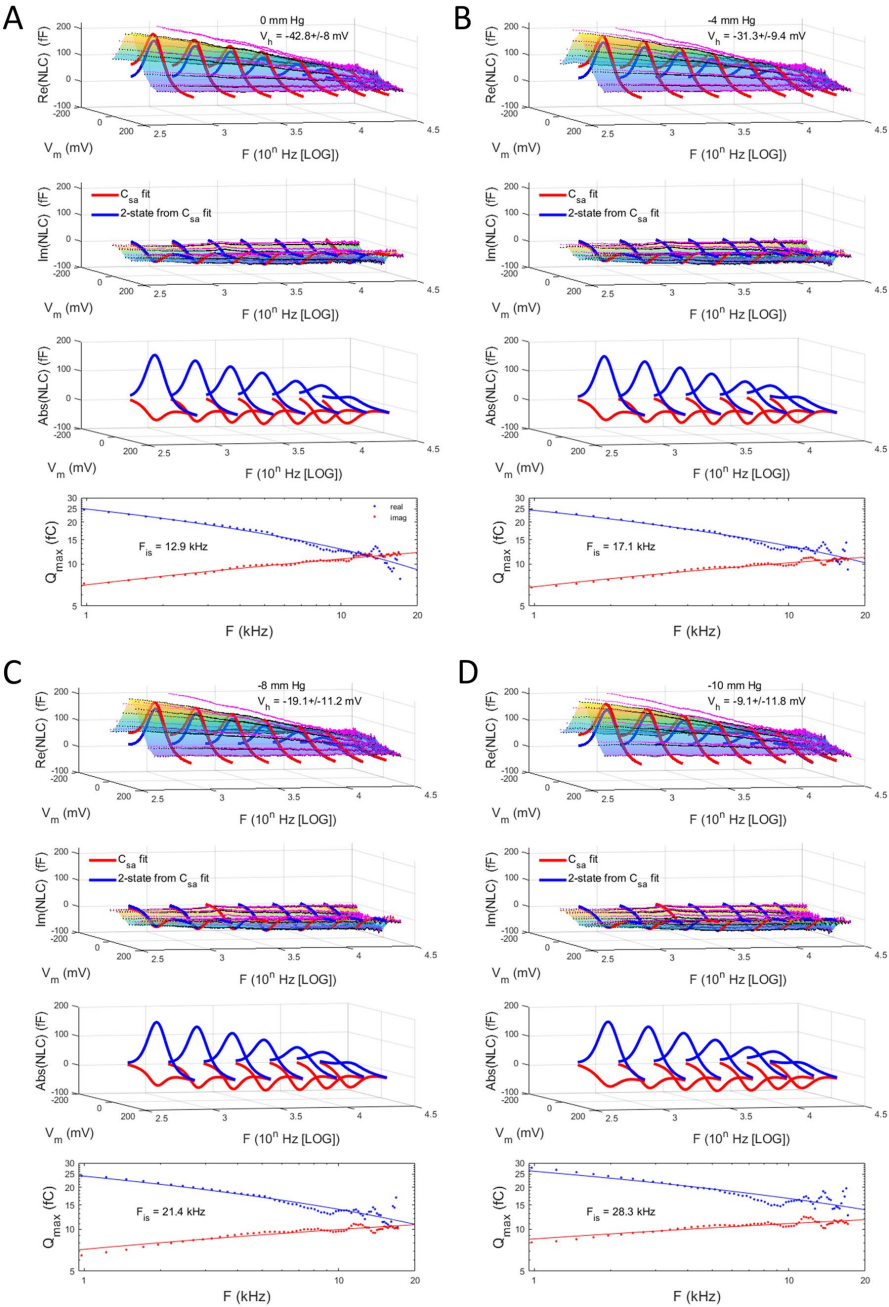
Effects of membrane tension on complex NLC. Pipette pressure was set to **(A)** 0 mmHg, **(B)** − 4 mmHg, **(C)** − 8 mmHg, and **(D)** − 10 mmHg. Responses are characterized as in Fig. 1 legend. The *bottom panels* show that F_is_ increases as tension increases.

Membrane tension is well known to shift V_h_ of OHC NLC. To investigate the influence of tension-induced V_h_ shift on real and imaginary components of NLC, we altered the tension delivered to the macro-patch membrane (Fig. 7). In 7 patches we were able to incrementally alter pipette pressure (thus, membrane tension) from 0 to − 2, − 4, − 6, − 8, and − 10 mm Hg (0 to − 1.22 kPa). Though substantial shifts in average V_h_ occur (from − 42.8 ± 8 to − 9.1 ± 11.8 mV), only slight changes in the real or imaginary components of complex NLC are readily visible. However, upon plotting intersecting frequencies (*lower panels*), clear changes in F_is_ are obvious. Increasing tension causes a shift in F_is_ to higher frequencies. For 0 mmHg, F_is_ is 12.9 kHz, whereas − 10 mmHg pressure shifts F_is_ to 28.3 kHz. Does initial V_h_ factor into these responses?

**Figure 8:**
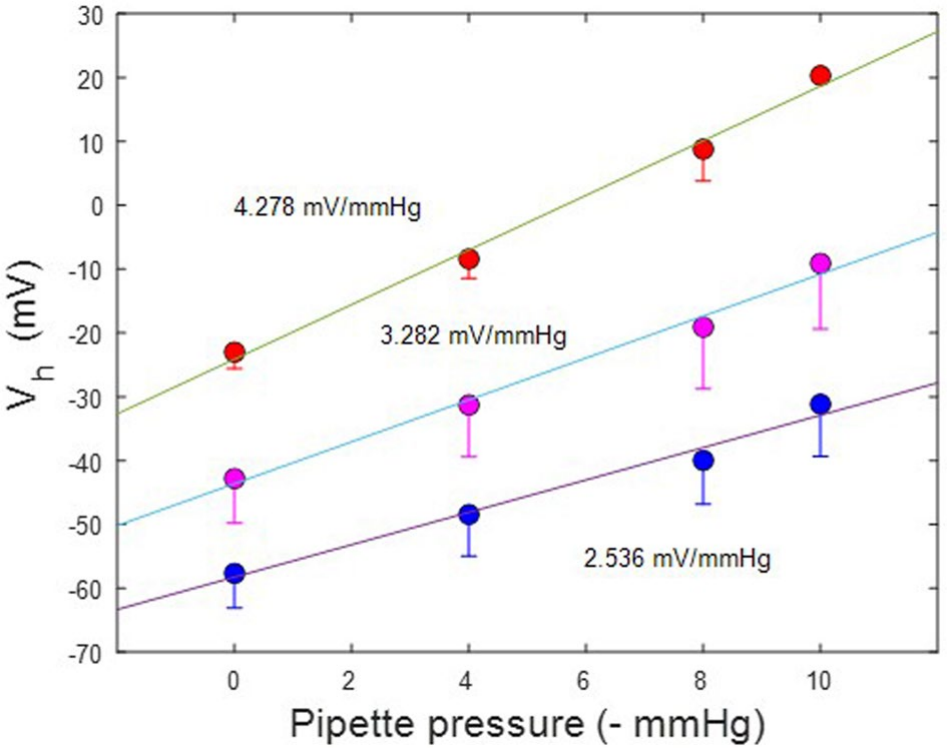
Shift in V_h_ due to tension versus initial V_h_. This plot shows that sensitivity to tension differs depending on initial V_h_. Upon categorization of initial V_h_ with values greater than and less than − 30 mV, differences in sensitivity to tension are exposed. In those patches with initial V_h_ greater than − 30 mV (red symbols; − 23.0 ± 2.6 mV; 4.28 mV/mmHg; n = 3), sensitivity is nearly double that of the group with initial V_h_ less than − 30 (blue symbols; − 57.6 ± 5.4 mV; 2.536 mV/mmHg; n = 4). For comparison, the relationship for all average patches is shown (magenta symbols), where a sensitivity to tensions is 3.3 mV/mmHg. This latter relationship is derived from data in Fig. 7. Linear fits provide the slope sensitivity.

As with our V_h_ categorization observations made above, we sought to determine the influence of initial V_h_ on tension effects. To this end, we categorized the patches into groups with initial V_h_ above and below − 30 mV. First, Fig. 8 illustrates that tension susceptibility depends on initial V_h_, with the positive group (red symbols; 4.28 mV/mmHg) showing V_h_ shift sensitivity nearly twice that of the negative group (blue symbols; 2.54 mV/mmHg). For all averaged patch responses (magenta symbols), the response was intermediate with standard errors the largest, as predicted from the categorization results.

**Figure 9:**
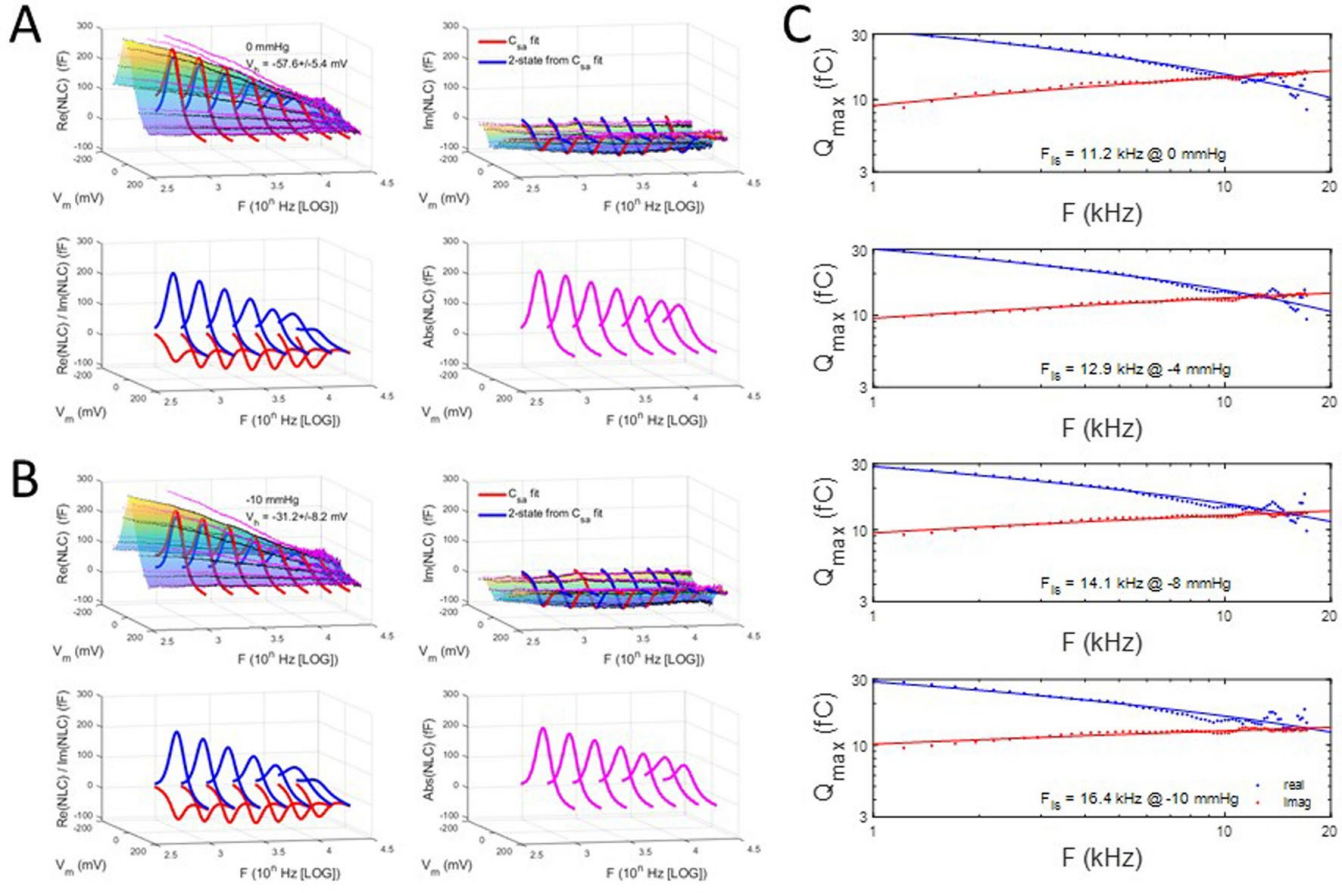
Membrane tension effects on the frequency response of complex capacitance for negative initial V_h_. **(A)** 0 mmHg, **(B)** − 10 mmHg. Clear changes, especially in the imaginary component, are noted between the two tensions. These changes are highlighted in **(C)** where the intersection frequency, F_is_, is found to *increase* with increases in tension. A shift from 11 to 16.2 kHz occurs between the two extremes.

In Fig. 9, we plot average NLC for the negative initial V_h_ group. Figure 9A shows results at 0 mmHg and Fig. 9B shows results at − 10 mmHg. Differences are apparent in the frequency responses, with Fig. 9C highlighting the influence of increasing membrane tension on the intersection frequency, F_is_. Increasing tension induces a shift in F_is_ to higher frequencies. For 0 mmHg, F_is_ is 11 kHz, whereas − 10 mmHg pressure shifts F_is_ to 16.2 kHz.

**Figure 10:**
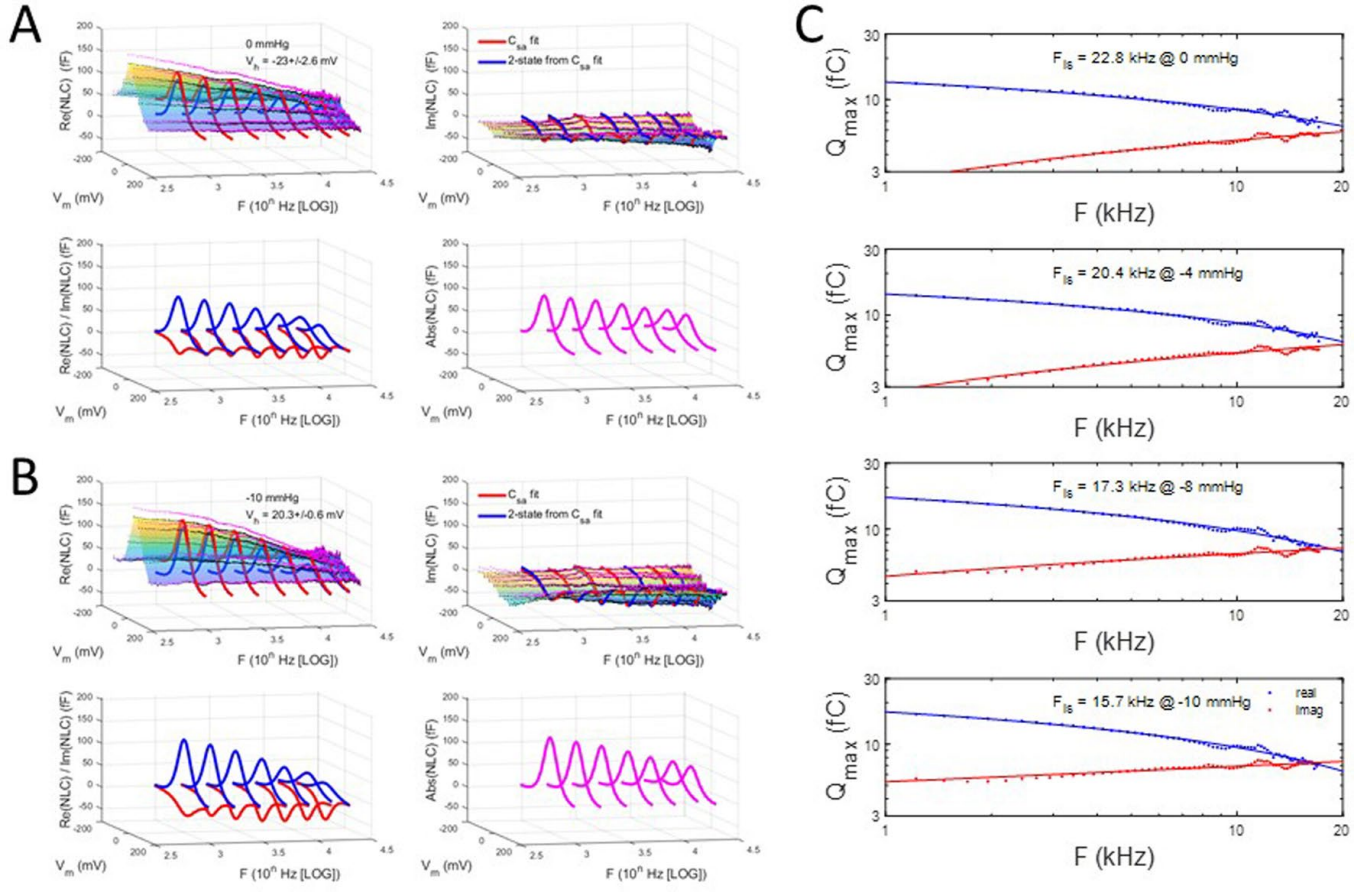
Membrane tension effects on the frequency response of complex capacitance for positive initial V_h_. **(A)** 0 mmHg, **(B)** − 10 mmHg. Clear changes, especially in the imaginary component, are noted between the two tensions. These changes are highlighted in **(C)** where the intersection frequency, F_is_, is found to *decrease* with increases in tension. A shift from 22.1 to 16.8 kHz occurs between the two extremes.

**Figure 11:**
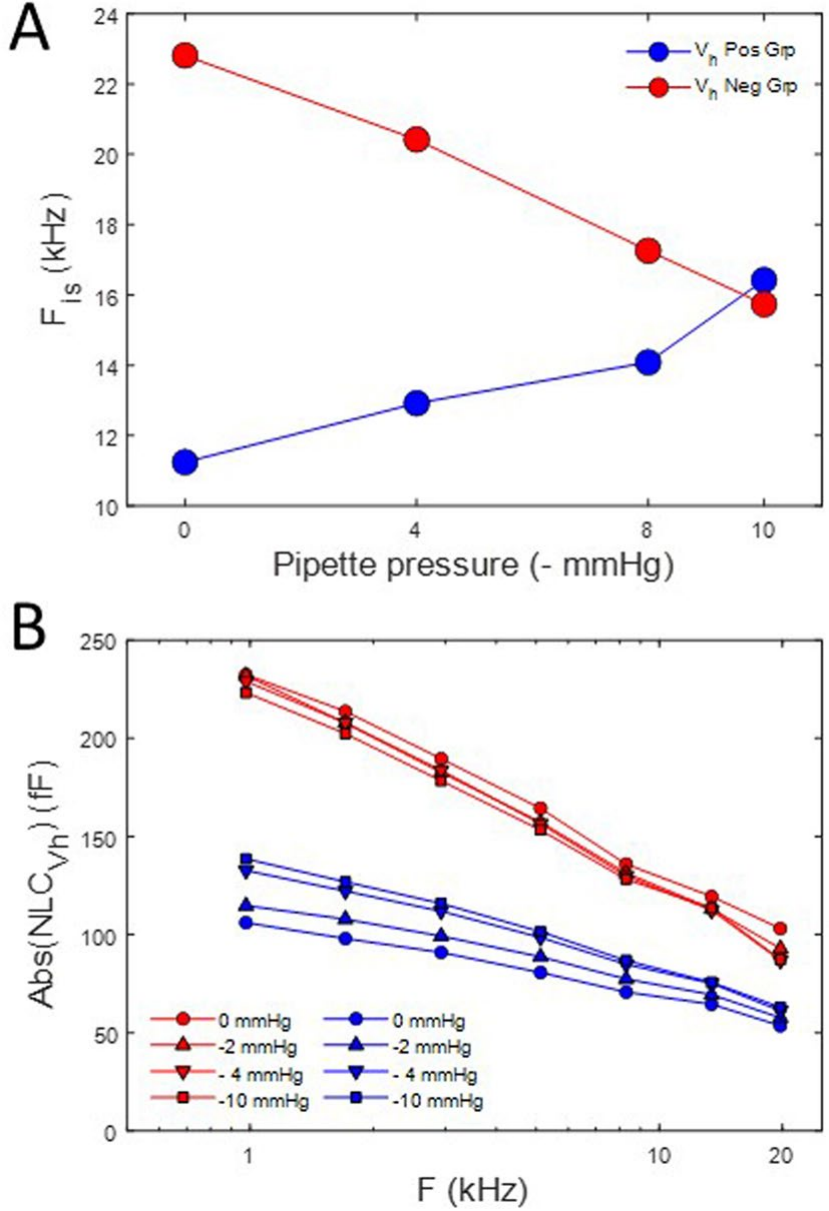
Summary of initial V_h_ influence on tension effects. **(A)** The direction of change in F_is_ is dependent on initial V_h_. Analyses were made with stray capacitance removal using either the + 160 mV (circles) or − 160 mV (asterisks) holding admittance. Results are identical, as expected. The two initial V_h_ group responses converge until a common F_is_ vs. tension is reached at − 10 mmHg, namely about 16 kHz. **(B)** Plot of the absolute magnitude of NLC for the two initial V_h_ groups. The negative group shows a very similar frequency response regardless of tension, but the positive group shows altered frequency responses, with increases in tension slowing the frequency response.

A similar analysis is shown in Fig. 10 for the positive initial V_h_ group. Surprisingly, in this case, increasing tension induces a shift in F_is_ to lower frequencies. The results for both groups are summarized in Fig. 11A. This plot shows that the two initial V_h_ group responses converge towards a common F_is_ at − 10 mmHg, namely about 16 kHz. It may not be a coincidence that this pressure value is close to the turgor pressure (~ 1 kPa) of the native OHC^38^. Finally, in Fig. 11B we plot the absolute magnitude of NLC for the two initial V_h_ groups. The negative group shows a very similar frequency response regardless of tension, and is in line with our previous observation on the immutable frequency response of the absolute magnitude of complex NLC during tension changes^7^. However, the positive group shows altered frequency responses, with increases in tension slowing the frequency response. Our data thus indicate an interaction between initial prestin state and tension sensitivity.

## Discussion

OHC NLC is frequency dependent and low pass. We first demonstrated this in guinea pig OHCs using AC voltage chirp stimuli to assess the real component of complex NLC nearly 30 years ago^13^. Over the years this has been confirmed across species^9,39–42^. Indeed, combining measures across a number of studies, we arrived at a collective estimate of NLC (absolute magnitude) frequency response that led us to suggest that prestin activity could not drive eM to sufficiently influence cochlear mechanics at very high acoustic frequencies (> 50 kHz), where cochlear amplification is expected to work best^7^. Recently, Rabbitt^21^, expanding on earlier modelling investigations^16,43–45^, has modelled OHC NLC as a piezoelectric process whose imaginary complex component takes on special significance, namely, signifying power output associated with prestin charge displacement. He modelled that as the real component of NLC decreases across frequency, the imaginary component increases—a frequency-dependent, reciprocal trade-off in magnitudes. Thus, he suggested that dielectric loss in voltage-driven prestin charge movement is indicative of considerable influence at high frequencies, thereby overriding our suggestions that prestin is limited in its high frequency effectiveness^7,46^.

Here we find experimentally that prestin’s complex NLC displays a partially reciprocal trade-off between real and imaginary components across interrogating frequency that follows predictions based on the PZE model of prestin^21^. However, we find that similar behavior is found with a simple 2-state kinetic model that is not piezoelectric, but solely voltage-dependent. Prior to Rabbitt’s work^21^, Iwasa^47^ developed a 2-state PZE model of prestin which explicitly incorporates tension dependence of prestin’s conformational states. Our 2-state model, which evidences real and imaginary parts of NLC, is dependent only on voltage, not tension, and thus is not piezoelectric. Thus, this type of complex behavior that we observe in our biophysical data is not necessarily due to piezoelectricity in prestin, and whether other predictions of the PZE model correspond to the observed prestin behavior that we find remains to be seen. To be sure, we are not claiming that prestin is not piezoelectric-like, since we have substantially contributed to the experimental evidence that it is, but only that non PZE models can generate real and imaginary components of NLC.

For decades, we and others^9,13,39,44,48^ have suggested that NLC and eM behavior are governed by the conformational kinetics of OHC motor (prestin) transitions, in line with traditional biophysical concepts of voltage-dependent protein behavior. Indeed, when we initially realized that prestin kinetics were influenced by chloride ions^49,50^, we suggested that delays introduced by stretched exponential transition rates in prestin would impact the phase of eM. Subsequently, we found that an eM phase lag (re voltage) develops across frequency and this could be influenced by chloride anions^51^. It is likely that the frequency dependent imaginary component of NLC we observe here correlates with that out of phase mechanical response. Thus, we agree with Rabbitt^21^ that the imaginary component of NLC may have special significance in prestin function, regardless of whether prestin works as a piezoelectric device or not. Nevertheless, our data show that absolute magnitude of NLC (representing total charge, including in and out of phase components) decreases as a power function of frequency, being 40 dB down at 77 kHz^7^. This is a minuscule fraction of voltage-driven prestin activity that exists at low frequencies. Based on PZE models^16,21,47^, mechanical loads can influence the frequency response of prestin. We agree that NLC and eM are susceptible to mechanical load, but the frequency response of whole-cell eM is slower than that of NLC, and likely corresponds to both cellular (external) and molecular (intrinsic) load components^8^. Thus, with minimal influence of external loads, we view our macro-patch data as providing the best estimate of prestin’s intrinsic conformational switching limit (including all influential molecular impedances), as originally espoused by Gale and Ashmore^39^.

Characteristics of OHC NLC have been known for some time to depend on initial conditions; for example, V_h_ depends on initial holding potential and anion binding is state-dependent^33,52,53^. In an effort to determine whether complex NLC shows sensitivity to initial conditions, we categorized our data into classes that had initial V_h_ values above and below − 30 mV. We found effects of initial conditions on the frequency response of real and imaginary components, where a metric of this relationship, F_is_, the intersection frequency of real and imaginary magnitude components varies with initial V_h_. As initial V_h_ shifts positively, F_is_ increases nonlinearly (see Fig. 6).

We also found initial V_h_ effects on the influence of static membrane tension on F_is_. Whereas the group with initial V_h_ positive to − 30 mV shows a decrease in F_is_ with increasing membrane tension, the opposite is found in the group with initial V_h_ negative to − 30 mV. At − 10 mmHg (1.22 kPa), each group’s F_is_ intersects near 16 kHz (see Fig. 11A). Interestingly, this pressure value is close to the turgor pressure (about 1 kPa) of the native cylindrical OHC^38^.

Lastly, we found that the group with initial V_h_ negative to − 30 mV shows little variation in the frequency response of the absolute magnitude of NLC with membrane tension, similar to what we observed previously^7^. However, the frequency response of the group with initial V_h_ positive to − 30 mV shows a decrease in the frequency response as membrane tension increases. From all these observations, it appears that the frequency response/magnitude of the imaginary component of NLC is mainly sensitive to initial V_h_. Though adherence to a minimum phase system response might predict a complimentary relationship between real and imaginary components, viscoelastic non-minimum phase systems are known in biology^54,55^. Consequently, our data indicate that the state of prestin, characterized by initial V_h_, influences frequency relationships between components of complex NLC, and thus, according to Rabbitt^21^, the power output of prestin activity. Nevertheless, changes in the frequency response are relatively small with our perturbations.

The fact that the individual components of complex NLC are differentially susceptible indicates that frequency-dependent phase differences occur between elements of sensor charge movement, possibly indicating that widely distributed sensor charge residues^56^ may independently interact within local intra-protein, nano-environments. Supporting this view, recent cryo-EM determined structural features of the mechano-sensitive channel MscS point to differential interactions of the protein with lipids surrounding and within the protein itself^57^. Thus, the study of complex NLC of our sensor charge residue mutations^56^ in HEK cell macro-patches, may assign particular residues responsible for the complex component responses. For example, we may find that certain mutations may remove the imaginary component without altering the real component.

As we noted above, there are several factors that influence V_h_. In the case of membrane tension, what could govern V_h_? There are numerous descriptions of membrane proteins/channels that are influenced by membrane tension^58^, with some possessing voltage-dependence; yet, interestingly, none have been deemed piezoelectric, per se. Rather, standard biophysical influences of load on a protein’s conformational state are espoused. For example, some Kv channels are voltage and tension sensitive, and simple models where changes in the equilibrium constant for channel opening, or equivalently the ratio of forward to backward transition rates, can account for tension effects on channel activity^59^. What other influences could there be in the absence of direct PZE effects or direct biophysical mechanical influences on prestin?

Several ideas come to mind. Perhaps the degree of cooperativity among prestin units alters with membrane tension. We recently provided evidence for negative cooperativity in prestin that was related to the density of prestin within the membrane^60^. Additionally, we previously modelled initial voltage influences on NLC as resulting from OHC molecular motor-motor (prestin) interactions^33^. For Kv 7.4 channels, cooperativity has been found to impart mechanical sensitivity to the channel in OHCs^61^. Another possibility is the presence of a conductive element influencing the interaction between voltage sensor charge and the membrane field, where that element alters with tension. Under this circumstance, the voltage sensed by the prestin charge may change during alterations in tension, leading to apparent V_h_ shifts relative to voltage clamp commands. Could this be a viscoelastic coupled conductive element, thus providing time dependent changes in voltage sensed? We have observed multi-exponential time-dependent behavior in NLC at fixed voltages^32,33^. Is G_metL_, the tension-dependent prestin leakage conductance we have identified^62^, the conductive component? We are currently testing complex NLC in prestin mutants that have reduced G_metL_ conductance.

Finally, the shift in V_h_ could additionally result from changes in the membrane surface charge or dipole potential that may accompany changes in membrane tension. Warshaviak et al.^63^ have found that physiologically relevant changes in membrane tension could shift that potential by tens of millivolts. Such effects could produce changes in the distribution of voltage-dependent protein conformations that would be evident as altered V_h_, despite effective voltage clamp. Interestingly, changes in temperature could conceivably alter the membrane dipole (in addition to many other properties of the membrane), since electrical breakdown in membrane is temperature dependent^64^. Thus, our observation of shifts in V_h_ due to temperature^29–31^ could also be independent of direct action on prestin. Of course, in this case of these “charge screening” effects, alterations in transition rates would not underlie V_h_ shifts, so this is unlikely to fully account for prestin’s tension responsiveness since we do find changes in the frequency response of real and imaginary components of complex NLC. Hence, transition rates are indeed altered.

To summarize our observations, we find that prestin’s complex NLC displays partial reciprocal trade-offs in real and imaginary component magnitude across frequency. The trade-off is prestin state-dependent in that the frequency response of the components alter with initial V_h_ and membrane tension. However, these observations do not nullify our initial observations^7,46^ that absolute complex NLC, signifying total charge moved, decreases precipitously as a power function of frequency; thus, prestin charge displacement that correlates with electromotility^9^ is expected to have limited physiological influence at very high frequencies in excess of 50 kHz.

Some final comments on the OHC’s ability to drive cochlear amplification at very high frequencies in a hypothetical cycle-by-cycle manner are in order. The membrane RC filter, which limits high frequency AC receptor potential generation, has been considered a potential problem for decades^65,66^, and while there are a number of publications that have suggested that the problem could be alleviated^17,67–69^, none have been directly tested in vivo. Thus, the RC problem may remain, and indeed arguments have been made for its effect on eM measured in vivo^10^. Both the potential RC problem and the low-pass nature of prestin charge movement pose serious obstacles for proposed theoretical mechanisms that offer to circumvent an OHC speed limit^8^.

## Methods

Detailed methods, including specifics of our voltage chirp stimulus protocol and extraction of complex NLC from patch admittance, are available in Refs.^7,46^. Briefly, extracellular solution was (in mM): NaCl 100, TEA-Cl 20, CsCl 20, CoCl_2_ 2, MgCl_2_ 1, CaCl_2_ 1, Hepes 10, pH 7.2. Experiments were performed at room temperature. Extracellular solution was in the patch pipette. On-cell macro-patches on the guinea pig OHC lateral membrane were made near the middle of the cylindrical cell, where prestin resides at high density^70,71^. Pipette inner tip size was about 3.5 μm, series resistance (R_s_) estimated to be below 1 MΩ, and seals at 0 mV near 5 GΩ (see Ref.^7^). Under these conditions, R_s_ effects are minimal^7^ and were not corrected for in this report. Furthermore, in this report we make relative assessments of NLC among groups of macro-patches. Chirp voltage stimuli (10 mV pk) were superimposed on step holding potentials from − 160 to + 160 mV, in 40 mV increments. As detailed previously^7^, the FFT derived admittance at + 160 mV, where NLC is absent, is subtracted from admittance at all other step potentials, thereby removing stray capacitance. Additionally, because we focus on effects of V_h_ on complex NLC behavior, we confirmed that subtractions for stray capacitance using either + 160 or − 160 mV responses give the exact same Boltzmann fits, as expected since stray capacitance will not influence prestin charge movement/NLC. A residual DC nonlinear conductance was also removed. DC conductance was determined from the DC component of FFT current response at each stepped voltage. Conductance, ΔI(0)/ΔV_hold_, was gauged by spline interpolating between each step voltage response, and differentiating digitally. Since, such an approach can have untoward effects at the end point voltage extremes, we fit the conductance linearly between 0 and 396 Hz (conductance at each frequency determined from the real components), and used the resultant fitted conductance at zero Hz for subtraction of the real components of admittance across frequencies. Figure 3 illustrates, in a simple 2-state kinetic model with solely voltage-dependent transition rates, the necessity of such nonlinear DC conductance subtractions to validly extract the imaginary component of NLC at low frequencies. The model is fully described in the “Results” Section. Patch membrane tension was imposed by changing pipette pressure. All data collection and analyses were performed with the software programs jClamp (www.scisoftco.com) and Matlab (www.mathworks.com). All means and standard errors (SE) are from individually analyzed patch data. Plots were made in Matlab.

In order to extract Boltzmann parameters, capacitance–voltage data were fit to the first derivative of a two-state Boltzmann function^13,39^, with an component of capacitance that characterizes sigmoidal changes in specific membrane capacitance^24,72^. We refer to this as the “C_sa_ fit” in text and figures.

1 $$\begin{array}{c}{C}_{m}=NLC {+{{C}_{sa}+C}_{\text{lin}}= Q}_{\text{max}}\frac{\text{ze}}{{k}_{B}T}\frac{b}{{\left(1+b\right)}^{2}}+{{C}_{sa}+C}_{\text{lin}},\end{array}$$

where $$b=exp\left(-ze\frac{{V}_{m}-{V}_{h}}{{k}_{B}T}\right)$$, $${C}_{sa}= \frac{{\Delta C}_{sa}}{(1+{b}^{-1})}.$$

Q_max_ is the maximum nonlinear charge moved, V_h_ is voltage at peak capacitance or equivalently, at half-maximum charge transfer, V_m_ is membrane potential, *z* is valence, C_lin_ is linear membrane capacitance, e is electron charge, *k*_*B*_ is Boltzmann’s constant, and T is absolute temperature. C_sa_ is a component of capacitance that characterizes sigmoidal changes in specific membrane capacitance, with ΔC_sa_ referring to the maximal change at very negative voltages^24,72^.

Qmax is the maximum nonlinear charge moved, Vh is voltage at peak capacitance or equivalently, at half-maximum charge transfer, Vm is membrane potential, z is valence, Clin is linear membrane capacitance, e is electron charge, kB is Boltzmann’s constant, and T is absolute temperature. Csa is a component of capacitance that characterizes sigmoidal changes in specific membrane capacitance, with ΔCsa referring to the maximal change at very negative voltages^24,72^.

For some data, a power fit as a function of frequency (*f*) was performed^46,73^.

2 $$C\left( f \right) = \, C_{0} + \, a \, \times \, f^{b} ,$$

where C_0_ is the asymptotic component, and *a* and *b* control the frequency response.
